# Crystal structure of aqua­bis­(hepta­fluoro­butano­ato-κ*O*)(1,10′-phenanthroline-κ^2^ *N*,*N*′)copper(II)

**DOI:** 10.1107/S2056989015022720

**Published:** 2016-01-01

**Authors:** Ibrahim Kani

**Affiliations:** a Anadolu University, Faculty of Sciences, Department of Chemistry, 26470 Eskişehir, Turkey

**Keywords:** crystal structure, copper(II) complex, hepta­fluoro­butanoic acid, *o*-phenanthroline, hydrogen bonding

## Abstract

The title compound, [Cu(C_4_F_7_O_2_)_2_(C_12_H_8_N_2_)(H_2_O)], is mononuclear and contains a penta­coordinated Cu^II^ ion. The geometry of Cu^II^ ion can be described as distorted square-pyramidal with two O atoms of two butano­ate anions and two N atoms of the *o*-phenanthroline ligand occupying the basal plane, and a water O atom located at the axial position. In the crystal, C—H⋯(O,F) and O—H⋯(O,F) hydrogen bonds and π–π inter­actions [centroid-to-centroid distance 3.533 (2) Å] link the mol­ecules into a three-dimensional supra­molecular structure.

## Abstract 2

The mol­ecular structure of a mononuclear Cu^II^ complex coordinated by hepta­fluoro­butanoic acid, 1,10-phenanthroline and a water mol­ecule is described.

## Chemical context   

Over the past decades, vast efforts have been dedicated to the rational design and synthesis of metal-carboxyl­ate coordination polymers due to their potential applications in medicine, electronics, magnetism, catalysis, gas storage, *etc* (Ahmad *et al.*, 2014 ▸; Patel *et al.*, 2013 ▸). In addition, metal–*o*-phenanthroline complexes and their derivatives have attracted much attention because of their unusual features (Ma *et al.*, 2004 ▸; Bi *et al.*, 2004 ▸; Wall *et al.*, 1999 ▸; Naing *et al.*, 1995 ▸). This work reports a new copper coordination complex, [Cu(C_4_F_7_O_2_)_2_(C_12_H_8_N_2_)(H_2_O)], resulting from the reaction of hepta­fluoro­butanoic acid and Cu^II^ ions in the presence of *o*-phenanthroline.
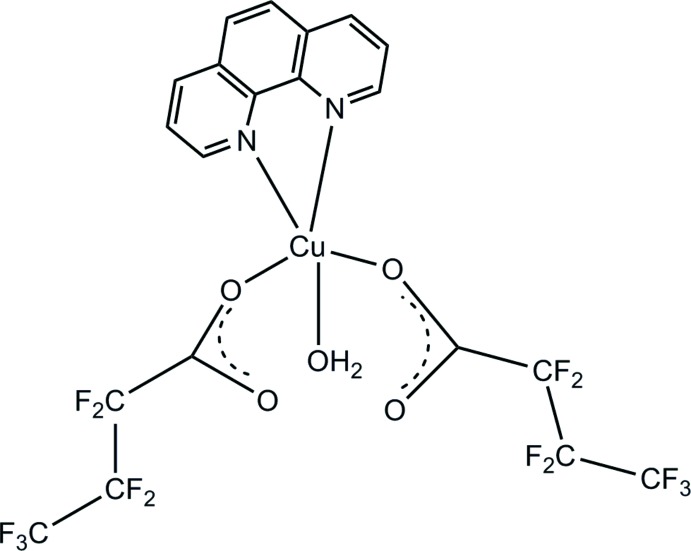


## Structural commentary   

**Figure 1:**
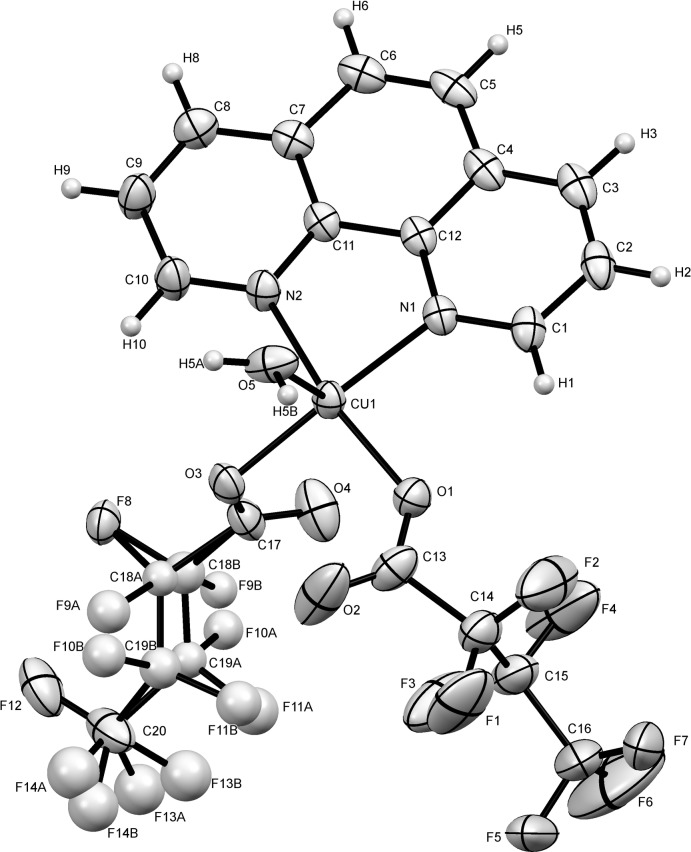
The mol­ecular structure of title compound, with displacement ellipsoids shown at the 30% probability level.

The neutral complex [Cu(C_4_F_7_O_2_)_2_(C_12_H_8_N_2_)(H_2_O)] is composed of a central Cu^II^ ion, coordinated by two oxygen atoms (O1 and O3) of two butano­ate anions, an oxygen atom (O5) of the water mol­ecule, and two nitro­gen atoms (N1 and N2) of the *N*,*N*′–chelating *o*–phenanthroline ligand (Fig. 1 ▸). Selected geometric parameters are presented in Table 1 ▸. The coordination about the Cu^II^ ion is better described as a square-pyramid. The geometry parameter τ, which is defined as τ = (β − α)/60, is applicable to five-coordinate structures within the structural continuum between trigonal–bipyramidal and tetra­gonal or rectangular pyramidal. For perfect tetra­gonal symmetry, τ is zero, and for perfect trigonal–bipyramidal geometry, τ becomes 1.0 (Addison *et al.*, 1984 ▸). In the title compound, the largest angles within the four atoms N1, N2, O2, O3 are β = 169.16 (12)° for O1–Cu1—N2, and α = 156.71 (11)° for N1—Cu1—O3. Thus, τ is 0.21, indicating a 79% rectangular pyramidal geometry.

The Cu—O bonds [1.942 (3) and 1.980 (3) Å] in the quadrilateral plane are shorter than the apical position [2.173 (3) Å]. The mean Cu—N(phen) distance of 2.043 Å and the bite angle N1—Cu1—N2 of 81.75 (12)° are close to the corresponding values observed in related copper–*o*-phenanthroline compounds (Beghidja *et al.*, 2014 ▸; Awaleh *et al.*, 2005 ▸). The cisoid bond angles are in the range 81.75 (12)–96.11 (11)°, and transoid ones are 156.71 (11)°, and 169.16 (12)° exhibiting substantial deviations from 90 and 180° for a square. These are consistent with literature values (Jing *et al.*, 2011 ▸). An intra­molecular C1—H1⋯O1 hydrogen bond occurs.

## Supra­molecular features   

**Figure 2:**
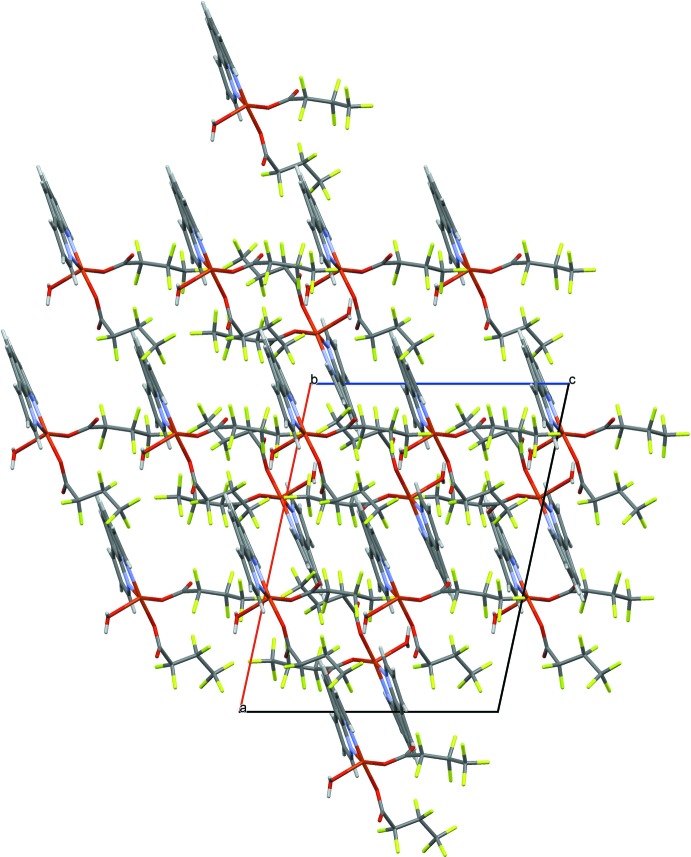
A partial view of the packing of the title complex, showing the formation of a hydrogen-bond pattern as well as edge-fused 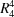
(16) rings. [Symmetry code: −*x* + 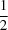, −*y* + 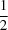, −*z*.]

**Figure 3:**
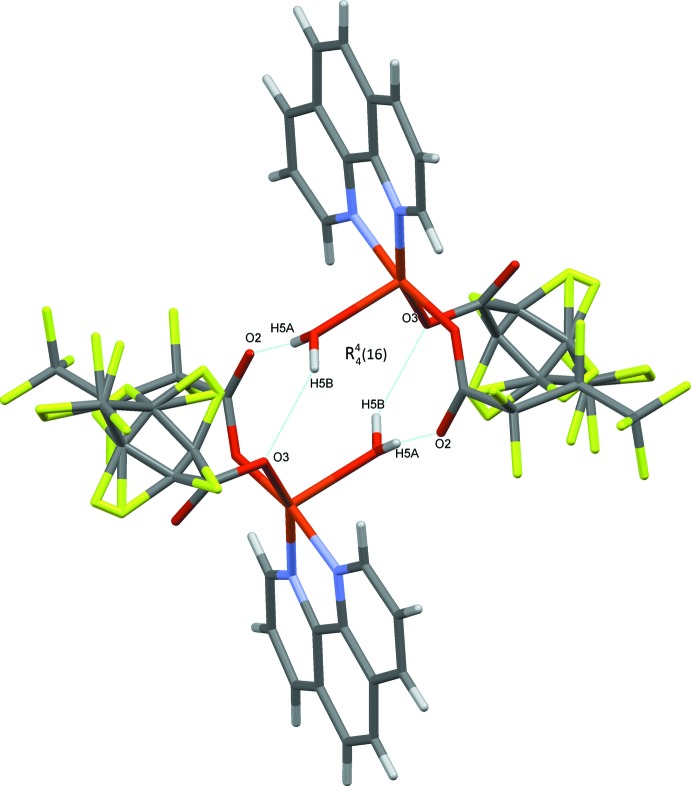
Representative O—H⋯O, C—H⋯O and C—H⋯F and π–π stacking inter­actions viewed along the *c* axis are drawn as dotted lines.

In the crystal, inter­molecular O—H⋯O, C—H⋯O and C—H⋯F hydrogen bonds (Table 2 ▸) link the mol­ecules into a three-dimensional network (Fig. 2 ▸). The oxygen atom (O5) of the water mol­ecule acts as a hydrogen-bond donor, *via* atoms H5*A* and H5*B*, to oxygen atom O3 of one coordinating carboxyl­ate group (−*x* + 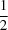, −*y* + 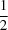, −*z*) and to the dangling oxygen atom O2 of the other coordinating carboxyl­ate group (−*x* + 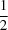, −*y* + 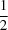, −*z*), thus enclosing centrosymmetric 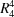
(16) ring motifs (Bernstein *et al.*, 1995 ▸) running parallel to the *b*-axis direction (Fig. 3 ▸). In addition, C—H⋯F and O—H⋯F hydrogen bonds are formed, (C6—H6⋯F4 and O5—H5*B*⋯F10; Table 2 ▸; Fig. 3 ▸); the H⋯F distances are comparable with those reported for C—H⋯F inter­actions (2.44–2.90 Å; Dunitz & Taylor *et al.*, 1997 ▸, Bianchi *et al.*, 2003 ▸; Lee *et al.*, 2000 ▸).

**Figure 4:**
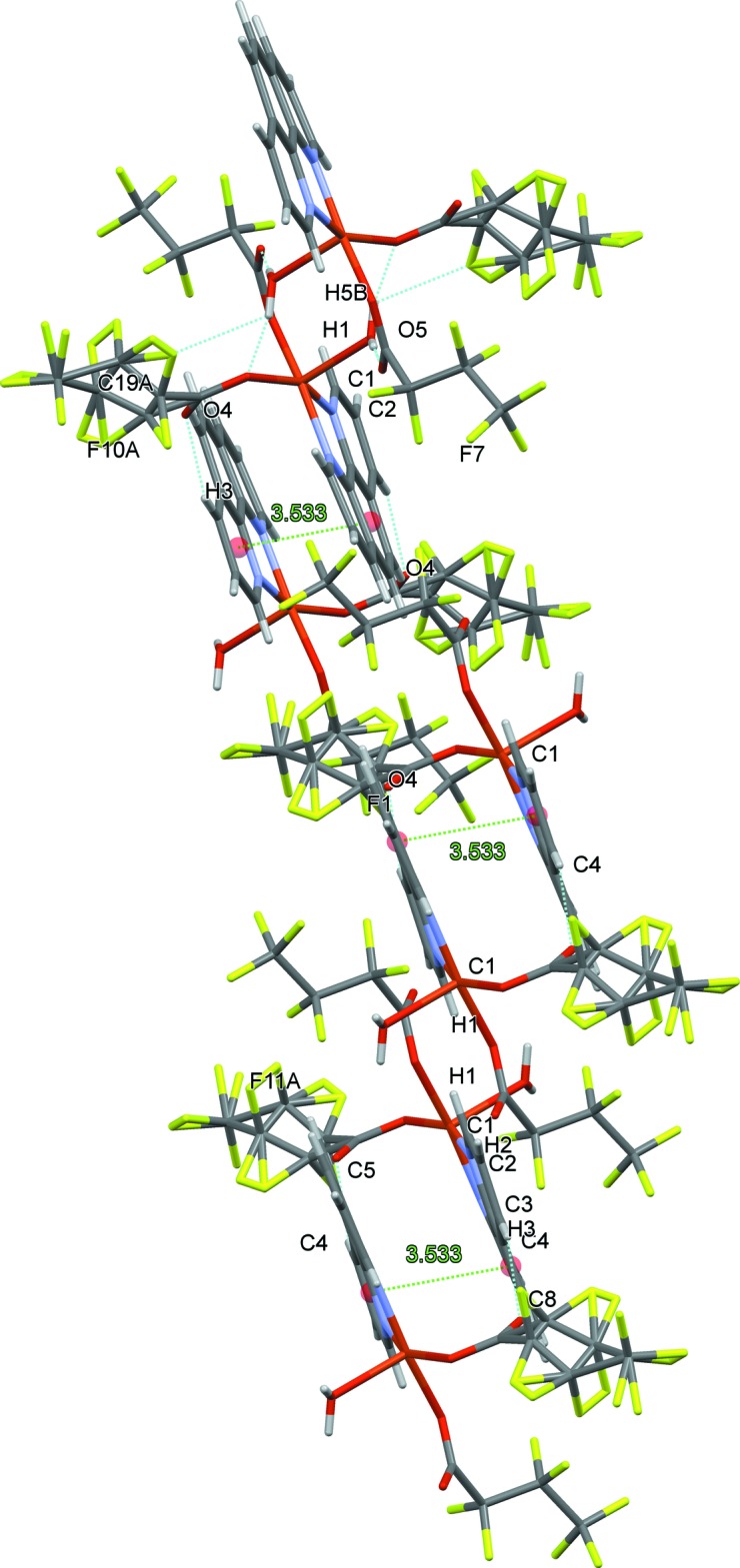
π–π interactions in the title compound.

In the crystal, the packing appears to be influenced by π–π stacking inter­actions between *o*-phenanthroline ring systems of neighboring mol­ecules, with the distance between the centroids of the N1/C1–C4/C12 and C4–C7/C11/C12 rings being 3.533 (2) Å. (Fig. 4 ▸). The shortest Cu⋯Cu distance in the supra­molecular structure is 7.845 Å.

## Database survey   

For hepta­fluoro­butanoic acid, see: Sokolov *et al.* (2011 ▸); Awaleh *et al.* (2005 ▸); King *et al.* (2009 ▸). For related structures and *o*-phenanthroline, see: Beghidja *et al.* (2014 ▸); Awaleh *et al.* (2005 ▸); Huang *et al.* (2010 ▸); Liu *et al.* (2010 ▸); Jing *et al.* (2011 ▸); Ma *et al.* (2004 ▸); Ni *et al.* (2011 ▸); Meundaeng *et al.* (2013 ▸); Sokolov *et al.* (2011 ▸); Yin *et al.* (2011 ▸).

## Synthesis and crystallization   

Cu(ClO4)·6H_2_O in methanol (0.076 mmol, 0.19 g) was added to a solution of *o*-phenanthroline (0.076 mmol, 0.14 g) and hepta­fluoro­butanoic acid (0.0160 mmol, 0.1ml) in methanol (7 ml). Afterwards the obtained transparent blue solution was left to evaporate slowly in the air at ambient temperature and after two weeks, X-ray quality crystals appeared as blue plates. They were filtered off, washed with diethyl ether and dried in the air. Yield: 46 mg, 86%.

## Refinement   

Crystal data, data collection and structure refinement details are summarized in Table 3 ▸. C-bound H atoms were placed in calculated positions and refined as riding with C—H = 0.95 Å and *U*_iso_(H) = 1.2*U*_eq_(C). The coordinates of the water H atoms were refined, and *U*_iso_(H) was set to be 2*U*_eq_(O). One of the hepta­fluoro­butano­ate groups is disordered over two sets of sites in a 0.705 (9):0.955 (9) ratio. Atoms associated with the disorder were refined with isotropic displacement parameters.

## Supplementary Material

Crystal structure: contains datablock(s) I. DOI: 10.1107/S2056989015022720/pj2026sup1.cif

Structure factors: contains datablock(s) I. DOI: 10.1107/S2056989015022720/pj2026Isup2.hkl

CCDC reference: 1434356

Additional supporting information:  crystallographic information; 3D view; checkCIF report

## Figures and Tables

**Table 1:** Selected geometric parameters (Å, °)

Cu1—O1	1.942 (3)	Cu1—N1	2.019 (3)
Cu1—O3	1.980 (3)	Cu1—O5	2.173 (3)
Cu1—N2	2.007 (3)		
			
O1—Cu1—O3	96.11 (11)	N2—Cu1—N1	81.75 (12)
O1—Cu1—N2	169.16 (12)	O1—Cu1—O5	97.20 (12)
O3—Cu1—N2	90.37 (12)	O3—Cu1—O5	96.84 (12)
O1—Cu1—N1	88.94 (11)	N2—Cu1—O5	90.61 (12)
O3—Cu1—N1	156.71 (11)	N1—Cu1—O5	105.09 (12)

**Table 2:** Hydrogen-bond geometry (Å, °)

*D*—H⋯*A*	*D*—H	H⋯*A*	*D*⋯*A*	*D*—H⋯*A*
C3—H3⋯O4^i^	0.95	2.33	3.196 (5)	151
C6—H6⋯F4^i^	0.95	2.54	3.217 (5)	128
O5—H5*B*⋯F10^ii^	0.84 (2)	2.45 (6)	2.931 (6)	117 (5)
O5—H5*B*⋯O3^ii^	0.84 (2)	2.31 (5)	2.881 (4)	125 (4)
O5—H5*A*⋯O2^ii^	0.84 (2)	1.87 (2)	2.707 (5)	175 (6)
C1—H1⋯O1	0.95	2.49	2.974 (5)	111

Symmetry codes: (i) 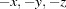; (ii) 
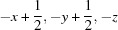
.

**Table 3:** Experimental details

Crystal data	
Chemical formula	[Cu(C_4_F_7_O_2_)_2_(C_12_H_8_N_2_)(H_2_O)]
*M*_r_	687.84
Crystal system, space group	Monoclinic, *C*2/*c*
Temperature (K)	110
*a*, *b*, *c* (Å)	18.0213 (5), 19.4619 (6), 13.8664 (4)
β (°)	102.205 (1)
*V* (Å^3^)	4753.4 (2)
*Z*	8
Radiation type	Mo *K*α
μ (mm^−1^)	1.07
Crystal size (mm)	0.35 × 0.26 × 0.20

Data collection	
Diffractometer	Bruker APEXII CCD area-detector
Absorption correction	Multi-scan (*SADABS*; Bruker, 2004 ▸)
*T*_min_, *T*_max_	0.707, 0.815
No. of measured, independent and observed [*I* > 2σ(*I*)] reflections	22348, 5892, 4467
*R*_int_	0.030
(sin θ/λ)_max_ (Å^−1^)	0.668

Refinement	
*R*[*F* ^2^ > 2σ(*F* ^2^)], *wR*(*F* ^2^), *S*	0.056, 0.156, 0.95
No. of reflections	5892
No. of parameters	450
No. of restraints	21
H-atom treatment	H atoms treated by a mixture of independent and constrained refinement
Δρ_max_, Δρ_min_ (e Å^−3^)	1.59, −1.08

Computer programs: *APEX2* and *SAINT* (Bruker, 2007 ▸), *SHELXS97*, *SHELXL97* and *SHELXTL* (Sheldrick, 2008 ▸) and *WinGX* (Farrugia, 2012 ▸).

## supplementary crystallographic information

### Crystal data

**Table:**

[Cu(C_4_F_7_O_2_)_2_(C_12_H_8_N_2_)(H_2_O)]	*F*(000) = 2712
*M**_r_* = 687.84	*D*_x_ = 1.922 Mg m^−^^3^
Monoclinic, *C*2/*c*	Mo *K*α radiation, λ = 0.71073 Å
*a* = 18.0213 (5) Å	Cell parameters from 6696 reflections
*b* = 19.4619 (6) Å	θ = 2.3–27.3°
*c* = 13.8664 (4) Å	µ = 1.07 mm^−^^1^
β = 102.205 (1)°	*T* = 110 K
*V* = 4753.4 (2) Å^3^	Plate, green
*Z* = 8	0.35 × 0.26 × 0.20 mm

### Data collection

**Table:**

Bruker APEXII CCD area-detector diffractometer	5892 independent reflections
Radiation source: fine-focus sealed tube	4467 reflections with *I* > 2σ(*I*)
Graphite monochromator	*R*_int_ = 0.030
phi and ω scans	θ_max_ = 28.3°, θ_min_ = 2.1°
Absorption correction: multi-scan (*SADABS*; Bruker, 2004)	*h* = −22→24
*T*_min_ = 0.707, *T*_max_ = 0.815	*k* = −25→25
22348 measured reflections	*l* = −18→18

### Refinement

**Table:**

Refinement on *F*^2^	Primary atom site location: structure-invariant direct methods
Least-squares matrix: full	Secondary atom site location: difference Fourier map
*R*[*F*^2^ > 2σ(*F*^2^)] = 0.056	Hydrogen site location: inferred from neighbouring sites
*wR*(*F*^2^) = 0.156	H atoms treated by a mixture of independent and constrained refinement
*S* = 0.95	*w* = 1/[σ^2^(*F*_o_^2^) + (0.0714*P*)^2^ + 30.9575*P*] where *P* = (*F*_o_^2^ + 2*F*_c_^2^)/3
5892 reflections	(Δ/σ)_max_ < 0.001
450 parameters	Δρ_max_ = 1.59 e Å^−^^3^
21 restraints	Δρ_min_ = −1.07 e Å^−^^3^

### Special details

**Table:**

Geometry. All e.s.d.'s (except the e.s.d. in the dihedral angle between two l.s. planes) are estimated using the full covariance matrix. The cell e.s.d.'s are taken into account individually in the estimation of e.s.d.'s in distances, angles and torsion angles; correlations between e.s.d.'s in cell parameters are only used when they are defined by crystal symmetry. An approximate (isotropic) treatment of cell e.s.d.'s is used for estimating e.s.d.'s involving l.s. planes.
Refinement. Refinement of *F*^2^ against ALL reflections. The weighted *R*-factor *wR* and goodness of fit *S* are based on *F*^2^, conventional *R*-factors *R* are based on *F*, with *F* set to zero for negative *F*^2^. The threshold expression of *F*^2^ > σ(*F*^2^) is used only for calculating *R*-factors(gt) *etc*. and is not relevant to the choice of reflections for refinement. *R*-factors based on *F*^2^ are statistically about twice as large as those based on *F*, and *R*- factors based on ALL data will be even larger.

### Fractional atomic coordinates and isotropic or equivalent isotropic displacement parameters (Å^2^)

**Table:**

	*x*	*y*	*z*	*U*_iso_*/*U*_eq_	Occ. (<1)
C1	0.1436 (2)	−0.00631 (18)	−0.0275 (3)	0.0331 (8)	
H1	0.1945	−0.0102	0.0084	0.040*	
C2	0.1051 (3)	−0.06556 (19)	−0.0674 (3)	0.0384 (9)	
H2	0.1301	−0.1088	−0.0597	0.046*	
C3	0.0312 (2)	−0.06099 (19)	−0.1176 (3)	0.0363 (8)	
H3	0.0045	−0.1012	−0.1439	0.044*	
C4	−0.0050 (2)	0.00320 (18)	−0.1299 (3)	0.0314 (7)	
C5	−0.0812 (2)	0.0146 (2)	−0.1834 (3)	0.0350 (8)	
H5	−0.1121	−0.0237	−0.2083	0.042*	
C6	−0.1098 (2)	0.0786 (2)	−0.1991 (3)	0.0358 (8)	
H6	−0.1600	0.0846	−0.2364	0.043*	
C7	−0.0660 (2)	0.13790 (19)	−0.1606 (3)	0.0310 (7)	
C8	−0.0907 (2)	0.2063 (2)	−0.1761 (3)	0.0377 (8)	
H8	−0.1395	0.2161	−0.2153	0.045*	
C9	−0.0445 (2)	0.2586 (2)	−0.1347 (3)	0.0417 (9)	
H9	−0.0604	0.3049	−0.1460	0.050*	
C10	0.0269 (2)	0.24334 (19)	−0.0752 (3)	0.0378 (8)	
H10	0.0581	0.2800	−0.0450	0.045*	
C11	0.00687 (19)	0.12756 (17)	−0.1035 (2)	0.0265 (7)	
C12	0.03820 (19)	0.06005 (17)	−0.0895 (2)	0.0261 (7)	
C13	0.3038 (2)	0.1216 (2)	0.1361 (3)	0.0446 (10)	
C14	0.3553 (3)	0.0667 (2)	0.1973 (3)	0.0497 (11)	
C15	0.3264 (3)	0.0430 (3)	0.2862 (3)	0.0532 (12)	
C16	0.3843 (4)	0.0061 (3)	0.3681 (4)	0.0755 (18)	
C17	0.1326 (2)	0.21520 (19)	0.1794 (3)	0.0378 (8)	
Cu1	0.15206 (2)	0.14635 (2)	0.01889 (3)	0.02752 (13)	
F1	0.42548 (17)	0.0915 (2)	0.2295 (3)	0.0965 (13)	
F2	0.3629 (3)	0.0143 (2)	0.1396 (3)	0.122 (2)	
F3	0.3026 (2)	0.0984 (2)	0.3269 (2)	0.1035 (16)	
F4	0.2709 (2)	−0.0012 (3)	0.2555 (4)	0.146 (3)	
F5	0.4354 (2)	0.04972 (17)	0.4146 (2)	0.0900 (13)	
F6	0.3516 (3)	−0.0217 (4)	0.4308 (4)	0.187 (3)	
F7	0.4236 (3)	−0.03979 (16)	0.3298 (3)	0.1140 (18)	
N1	0.11117 (17)	0.05511 (14)	−0.0382 (2)	0.0272 (6)	
N2	0.05191 (16)	0.17936 (15)	−0.0599 (2)	0.0296 (6)	
O1	0.23861 (16)	0.09849 (14)	0.09756 (19)	0.0376 (6)	
O2	0.33070 (19)	0.1784 (2)	0.1330 (4)	0.0850 (15)	
O3	0.15945 (15)	0.22857 (13)	0.1045 (2)	0.0358 (6)	
O4	0.1086 (2)	0.16038 (16)	0.2021 (3)	0.0548 (9)	
O5	0.21033 (16)	0.18964 (17)	−0.0891 (2)	0.0435 (7)	
C19A	0.1413 (3)	0.2694 (3)	0.3503 (4)	0.0340 (14)	0.705 (9)
C18A	0.1341 (3)	0.2831 (3)	0.2401 (4)	0.0308 (14)	0.705 (9)
F10	0.1905 (3)	0.3264 (2)	0.2301 (4)	0.0468 (12)	0.705 (9)
F10A	0.0794 (3)	0.2369 (3)	0.3662 (4)	0.0391 (11)	0.705 (9)
F11A	0.2022 (3)	0.2288 (2)	0.3824 (4)	0.0542 (13)	0.705 (9)
F13A	0.1512 (3)	0.3159 (3)	0.5086 (4)	0.0653 (15)	0.705 (9)
F14A	0.2185 (6)	0.3659 (6)	0.4174 (5)	0.067 (2)	0.705 (9)
C18B	0.1086 (7)	0.2638 (6)	0.2581 (8)	0.031 (3)	0.295 (9)
C19B	0.1812 (6)	0.2937 (6)	0.3221 (8)	0.033 (3)	0.295 (9)
F9B	0.0682 (7)	0.2367 (6)	0.3213 (9)	0.034 (2)	0.295 (9)
F10B	0.2100 (7)	0.3414 (6)	0.2688 (9)	0.040 (2)	0.295 (9)
F11B	0.2321 (6)	0.2427 (5)	0.3454 (9)	0.046 (3)	0.295 (9)
F13B	0.1619 (7)	0.2812 (7)	0.4850 (8)	0.055 (3)	0.295 (9)
F14B	0.2290 (11)	0.3614 (15)	0.4558 (11)	0.063 (5)	0.295 (9)
F8	0.06788 (13)	0.31602 (11)	0.20682 (16)	0.0373 (5)	
F12	0.10008 (19)	0.37634 (14)	0.3905 (2)	0.0641 (8)	
C20	0.1565 (3)	0.3330 (2)	0.4172 (3)	0.0571 (13)	
H5A	0.200 (3)	0.2313 (13)	−0.100 (5)	0.086*	
H5B	0.2577 (13)	0.185 (3)	−0.081 (5)	0.086*	

### Atomic displacement parameters (Å^2^)

**Table:**

	*U*^11^	*U*^22^	*U*^33^	*U*^12^	*U*^13^	*U*^23^
C1	0.043 (2)	0.0282 (17)	0.0332 (18)	0.0037 (15)	0.0187 (16)	0.0015 (14)
C2	0.060 (3)	0.0249 (17)	0.0364 (19)	0.0036 (16)	0.0247 (18)	−0.0016 (14)
C3	0.054 (2)	0.0292 (17)	0.0328 (18)	−0.0083 (16)	0.0249 (17)	−0.0060 (14)
C4	0.0391 (19)	0.0321 (17)	0.0289 (16)	−0.0094 (15)	0.0207 (15)	−0.0066 (14)
C5	0.039 (2)	0.042 (2)	0.0291 (17)	−0.0145 (16)	0.0179 (15)	−0.0131 (15)
C6	0.0314 (19)	0.049 (2)	0.0299 (17)	−0.0093 (16)	0.0124 (15)	−0.0076 (16)
C7	0.0293 (17)	0.0372 (19)	0.0297 (16)	−0.0024 (14)	0.0130 (14)	−0.0025 (14)
C8	0.0292 (18)	0.044 (2)	0.040 (2)	0.0023 (16)	0.0086 (15)	0.0013 (17)
C9	0.033 (2)	0.0312 (19)	0.059 (3)	0.0060 (15)	0.0050 (18)	0.0054 (18)
C10	0.0318 (19)	0.0266 (17)	0.054 (2)	−0.0013 (14)	0.0066 (17)	0.0010 (16)
C11	0.0274 (16)	0.0270 (15)	0.0287 (16)	−0.0049 (13)	0.0144 (13)	−0.0016 (13)
C12	0.0294 (17)	0.0266 (15)	0.0269 (15)	−0.0031 (13)	0.0165 (13)	−0.0029 (12)
C13	0.033 (2)	0.056 (3)	0.045 (2)	0.0097 (19)	0.0077 (17)	0.0139 (19)
C14	0.053 (3)	0.047 (2)	0.044 (2)	0.015 (2)	−0.0026 (19)	−0.0041 (19)
C15	0.049 (3)	0.052 (3)	0.049 (2)	−0.009 (2)	−0.012 (2)	0.015 (2)
C16	0.100 (5)	0.056 (3)	0.053 (3)	0.005 (3)	−0.025 (3)	0.008 (3)
C17	0.037 (2)	0.0278 (18)	0.048 (2)	−0.0001 (15)	0.0085 (17)	−0.0129 (16)
Cu1	0.0256 (2)	0.0237 (2)	0.0338 (2)	−0.00175 (16)	0.00763 (16)	0.00126 (16)
F1	0.0325 (15)	0.120 (3)	0.128 (3)	0.0171 (17)	−0.0034 (17)	0.051 (3)
F2	0.175 (4)	0.108 (3)	0.061 (2)	0.099 (3)	−0.027 (2)	−0.033 (2)
F3	0.115 (3)	0.151 (4)	0.0462 (17)	0.081 (3)	0.0195 (18)	0.009 (2)
F4	0.094 (3)	0.141 (4)	0.162 (4)	−0.081 (3)	−0.066 (3)	0.109 (3)
F5	0.110 (3)	0.065 (2)	0.0649 (19)	0.0228 (19)	−0.0494 (19)	−0.0231 (16)
F6	0.147 (5)	0.268 (8)	0.127 (4)	−0.009 (5)	−0.010 (4)	0.148 (5)
F7	0.171 (4)	0.0399 (17)	0.090 (3)	0.039 (2)	−0.064 (3)	−0.0144 (17)
N1	0.0322 (15)	0.0250 (13)	0.0279 (13)	−0.0003 (11)	0.0146 (12)	−0.0006 (11)
N2	0.0248 (14)	0.0259 (14)	0.0395 (16)	−0.0015 (11)	0.0097 (12)	0.0022 (12)
O1	0.0396 (15)	0.0344 (14)	0.0362 (13)	0.0061 (11)	0.0022 (11)	−0.0013 (11)
O2	0.0348 (18)	0.077 (3)	0.131 (4)	−0.0168 (17)	−0.010 (2)	0.060 (3)
O3	0.0354 (14)	0.0245 (12)	0.0457 (15)	−0.0064 (10)	0.0045 (12)	−0.0028 (11)
O4	0.064 (2)	0.0404 (16)	0.071 (2)	−0.0094 (15)	0.0411 (18)	−0.0060 (15)
O5	0.0326 (14)	0.0626 (19)	0.0336 (14)	−0.0162 (13)	0.0033 (12)	0.0108 (13)
C19A	0.036 (3)	0.022 (2)	0.043 (3)	−0.003 (2)	0.009 (2)	0.001 (2)
C18A	0.034 (3)	0.017 (2)	0.046 (3)	−0.007 (2)	0.017 (2)	−0.006 (2)
F10	0.060 (3)	0.031 (2)	0.060 (3)	−0.0265 (18)	0.036 (3)	−0.019 (2)
F10A	0.049 (3)	0.0278 (17)	0.046 (3)	−0.0078 (16)	0.022 (2)	0.004 (2)
F11A	0.046 (3)	0.044 (2)	0.068 (3)	0.0102 (19)	0.001 (2)	0.006 (2)
F13A	0.099 (4)	0.059 (3)	0.035 (2)	−0.008 (3)	0.007 (2)	−0.003 (2)
F14A	0.080 (4)	0.063 (4)	0.062 (5)	−0.034 (3)	0.020 (4)	−0.017 (5)
C18B	0.030 (7)	0.025 (7)	0.038 (7)	−0.002 (5)	0.007 (6)	0.005 (5)
C19B	0.039 (7)	0.032 (6)	0.027 (6)	−0.004 (5)	0.006 (5)	0.002 (5)
F9B	0.034 (5)	0.031 (4)	0.040 (6)	−0.006 (3)	0.015 (5)	0.002 (5)
F10B	0.052 (6)	0.031 (5)	0.045 (6)	−0.017 (4)	0.024 (5)	−0.007 (4)
F11B	0.032 (5)	0.045 (5)	0.055 (6)	0.006 (4)	−0.004 (4)	−0.006 (4)
F13B	0.065 (7)	0.072 (8)	0.028 (5)	−0.028 (6)	0.007 (4)	0.003 (5)
F14B	0.087 (12)	0.063 (8)	0.054 (10)	−0.021 (8)	0.046 (9)	−0.013 (10)
F8	0.0431 (13)	0.0271 (10)	0.0419 (12)	0.0077 (9)	0.0096 (10)	0.0026 (9)
F12	0.092 (2)	0.0404 (14)	0.0661 (18)	−0.0051 (15)	0.0298 (17)	−0.0202 (13)
C20	0.091 (4)	0.046 (2)	0.033 (2)	−0.024 (3)	0.010 (2)	−0.0066 (18)

### Geometric parameters (Å, º)

**Table:**

C1—N1	1.325 (4)	C15—C16	1.546 (7)
C1—C2	1.398 (5)	C16—F6	1.271 (8)
C1—H1	0.9500	C16—F5	1.316 (7)
C2—C3	1.369 (6)	C16—F7	1.319 (8)
C2—H2	0.9500	C17—O4	1.218 (5)
C3—C4	1.403 (5)	C17—O3	1.261 (5)
C3—H3	0.9500	C17—C18A	1.564 (6)
C4—C12	1.400 (5)	C17—C18B	1.572 (11)
C4—C5	1.433 (6)	Cu1—O1	1.942 (3)
C5—C6	1.348 (6)	Cu1—O3	1.980 (3)
C5—H5	0.9500	Cu1—N2	2.007 (3)
C6—C7	1.437 (5)	Cu1—N1	2.019 (3)
C6—H6	0.9500	Cu1—O5	2.173 (3)
C7—C11	1.397 (5)	O5—H5A	0.840 (19)
C7—C8	1.406 (5)	O5—H5B	0.843 (19)
C8—C9	1.362 (6)	C19A—F10A	1.341 (7)
C8—H8	0.9500	C19A—F11A	1.349 (6)
C9—C10	1.405 (5)	C19A—C18A	1.530 (7)
C9—H9	0.9500	C19A—C20	1.536 (7)
C10—N2	1.326 (5)	C18A—F8	1.348 (6)
C10—H10	0.9500	C18A—F10	1.349 (8)
C11—N2	1.354 (4)	F13A—C20	1.332 (6)
C11—C12	1.427 (5)	F14A—C20	1.288 (9)
C12—N1	1.360 (4)	C18B—F9B	1.359 (15)
C13—O2	1.211 (6)	C18B—F8	1.363 (11)
C13—O1	1.265 (5)	C18B—C19B	1.532 (12)
C13—C14	1.546 (6)	C19B—F11B	1.344 (12)
C14—F2	1.321 (5)	C19B—F10B	1.357 (13)
C14—F1	1.339 (6)	C19B—C20	1.665 (13)
C14—C15	1.508 (7)	F13B—C20	1.366 (11)
C15—F4	1.321 (6)	F14B—C20	1.417 (16)
C15—F3	1.329 (6)	F12—C20	1.313 (6)
			
N1—C1—C2	122.0 (4)	O3—Cu1—N2	90.37 (12)
N1—C1—H1	119.0	O1—Cu1—N1	88.94 (11)
C2—C1—H1	119.0	O3—Cu1—N1	156.71 (11)
C3—C2—C1	119.7 (4)	N2—Cu1—N1	81.75 (12)
C3—C2—H2	120.1	O1—Cu1—O5	97.20 (12)
C1—C2—H2	120.1	O3—Cu1—O5	96.84 (12)
C2—C3—C4	119.8 (3)	N2—Cu1—O5	90.61 (12)
C2—C3—H3	120.1	N1—Cu1—O5	105.09 (12)
C4—C3—H3	120.1	C1—N1—C12	118.5 (3)
C12—C4—C3	116.7 (3)	C1—N1—Cu1	129.3 (3)
C12—C4—C5	118.4 (3)	C12—N1—Cu1	112.0 (2)
C3—C4—C5	124.8 (3)	C10—N2—C11	118.4 (3)
C6—C5—C4	121.2 (3)	C10—N2—Cu1	128.6 (3)
C6—C5—H5	119.4	C11—N2—Cu1	113.0 (2)
C4—C5—H5	119.4	C13—O1—Cu1	129.1 (3)
C5—C6—C7	121.4 (4)	C17—O3—Cu1	109.7 (2)
C5—C6—H6	119.3	Cu1—O5—H5A	112 (4)
C7—C6—H6	119.3	Cu1—O5—H5B	120 (4)
C11—C7—C8	116.9 (3)	H5A—O5—H5B	108 (3)
C11—C7—C6	118.2 (3)	F10A—C19A—F11A	108.4 (5)
C8—C7—C6	124.9 (4)	F10A—C19A—C18A	110.4 (5)
C9—C8—C7	119.8 (4)	F11A—C19A—C18A	108.8 (5)
C9—C8—H8	120.1	F10A—C19A—C20	109.2 (5)
C7—C8—H8	120.1	F11A—C19A—C20	104.1 (4)
C8—C9—C10	119.5 (4)	C18A—C19A—C20	115.5 (4)
C8—C9—H9	120.2	F8—C18A—F10	107.4 (5)
C10—C9—H9	120.2	F8—C18A—C19A	107.8 (4)
N2—C10—C9	122.0 (4)	F10—C18A—C19A	107.7 (5)
N2—C10—H10	119.0	F8—C18A—C17	107.7 (4)
C9—C10—H10	119.0	F10—C18A—C17	113.6 (4)
N2—C11—C7	123.3 (3)	C19A—C18A—C17	112.3 (4)
N2—C11—C12	116.2 (3)	F9B—C18B—F8	108.9 (9)
C7—C11—C12	120.5 (3)	F9B—C18B—C19B	106.3 (10)
N1—C12—C4	123.3 (3)	F8—C18B—C19B	108.5 (9)
N1—C12—C11	116.6 (3)	F9B—C18B—C17	118.6 (9)
C4—C12—C11	120.1 (3)	F8—C18B—C17	106.5 (7)
O2—C13—O1	130.7 (4)	C19B—C18B—C17	107.7 (9)
O2—C13—C14	116.8 (4)	F11B—C19B—F10B	108.4 (11)
O1—C13—C14	112.5 (4)	F11B—C19B—C18B	108.3 (9)
F2—C14—F1	105.6 (5)	F10B—C19B—C18B	108.8 (11)
F2—C14—C15	110.9 (5)	F11B—C19B—C20	115.6 (9)
F1—C14—C15	107.6 (4)	F10B—C19B—C20	108.4 (9)
F2—C14—C13	109.1 (4)	C18B—C19B—C20	107.2 (8)
F1—C14—C13	110.4 (4)	C18A—F8—C18B	29.1 (5)
C15—C14—C13	112.9 (4)	F14A—C20—F12	107.5 (7)
F4—C15—F3	112.0 (5)	F14A—C20—F13A	111.0 (5)
F4—C15—C14	107.7 (4)	F12—C20—F13A	102.8 (5)
F3—C15—C14	107.3 (4)	F14A—C20—F13B	115.4 (8)
F4—C15—C16	106.4 (4)	F12—C20—F13B	127.3 (7)
F3—C15—C16	107.1 (4)	F13A—C20—F13B	34.1 (5)
C14—C15—C16	116.5 (5)	F14A—C20—F14B	22.1 (8)
F6—C16—F5	108.4 (6)	F12—C20—F14B	116.9 (13)
F6—C16—F7	110.6 (6)	F13A—C20—F14B	89.1 (8)
F5—C16—F7	104.8 (6)	F13B—C20—F14B	95.9 (11)
F6—C16—C15	111.3 (6)	F14A—C20—C19A	116.4 (6)
F5—C16—C15	110.7 (4)	F12—C20—C19A	108.6 (4)
F7—C16—C15	110.8 (5)	F13A—C20—C19A	109.6 (4)
O4—C17—O3	127.8 (3)	F13B—C20—C19A	78.5 (7)
O4—C17—C18A	124.6 (4)	F14B—C20—C19A	125.1 (12)
O3—C17—C18A	107.6 (4)	F14A—C20—C19B	81.5 (6)
O4—C17—C18B	100.5 (5)	F12—C20—C19B	113.1 (5)
O3—C17—C18B	131.1 (5)	F13A—C20—C19B	136.4 (6)
C18A—C17—C18B	25.1 (4)	F13B—C20—C19B	102.4 (8)
O1—Cu1—O3	96.11 (11)	F14B—C20—C19B	95.6 (10)
O1—Cu1—N2	169.16 (12)	C19A—C20—C19B	36.3 (4)

### Hydrogen-bond geometry (Å, º)

**Table:**

*D*—H···*A*	*D*—H	H···*A*	*D*···*A*	*D*—H···*A*
C3—H3···O4^i^	0.95	2.33	3.196 (5)	151
C6—H6···F4^i^	0.95	2.54	3.217 (5)	128
O5—H5*B*···F10^ii^	0.84 (2)	2.45 (6)	2.931 (6)	117 (5)
O5—H5*B*···O3^ii^	0.84 (2)	2.31 (5)	2.881 (4)	125 (4)
O5—H5*A*···O2^ii^	0.84 (2)	1.87 (2)	2.707 (5)	175 (6)
C1—H1···O1	0.95	2.49	2.974 (5)	111

Symmetry codes: (i) −*x*, −*y*, −*z*; (ii) −*x*+1/2, −*y*+1/2, −*z*.
